# Factors associated with non-adherence to polychemotherapy treatment for leprosy

**DOI:** 10.1590/0034-7167-2024-0437

**Published:** 2025-12-08

**Authors:** Johnne Silva da Cruz, Eliane Patricia Lino Pereira Franchi

**Affiliations:** I Fundação Escola de Saúde Pública de Palmas, Palmas, Tocantins, Brazil.

**Keywords:** Leprosy, Precipitating Factors, Medication Non-Adherence, Treatment Refusal, Primary Health Care, Lepra, Factores Desencadenantes, No Adherencia a la Medicação, Negativa del Paciente al Tratamiento, Atenação Primaria de Salud

## Abstract

**Objectives:**

to identify factors associated with non-adherence to polychemotherapy treatment for leprosy.

**Methods:**

a cross-sectional study, carried out with cases of abandonment of leprosy treatment in the municipality of Palmas-TO. The cases were identified from the Notification System for Notifiable Diseases, and a questionnaire was subsequently applied.

**Results:**

dropout rates for 2017 and 2018 were 8.3% and 3.12%, respectively. Forty people participated in the study: the majority were women (57.5%), with an average age of 48.2 years, 75% were black/brown, 50% had completed/incomplete elementary education, and 52.5% were retired/unemployed. Factors associated with adherence to leprosy treatment were lack of improvement in symptoms and having the diagnosis ruled out by another physician/not believing they had leprosy.

**Conclusions:**

the results point to the difficulty in accepting leprosy by cases and the importance of healthcare professionals in health education and adequate leprosy diagnosis.

## RESUMEN

**Objetivos:**

identificar factores asociados a la no adherencia al tratamiento poliquimioterapéutico para la lepra.

**Métodos:**

estudio transversal, realizado con casos de abandono del tratamiento de la lepra en el municipio de Palmas-TO. Los casos fueron identificados a partir del Sistema de Notificação de Enfermedades de Notificação Obligatoria y posteriormente se aplicó un cuestionario.

**Resultados:**

las tasas de deseração para los años 2017 y 2018 fueron de 8,3% y 3,12%, respectivamente. Participaron en el estudio cuarenta personas: la mayoría eran mujeres (57,5%), con una edad media de 48,2 años, el 75% eran de raza negra/mestizas, el 50% tenía educação primaria completa/incompleta y el 52,5% estaban jubilados/desempleados. Los factores asociados con la adherencia al tratamiento de la lepra fueron la falta de mejoría de los síntomas y el hecho de que otro médico descartara el diagnóstico o no creyera que se tenía la enfermedad.

**Conclusiones:**

los resultados apuntan a la dificultad en la aceptação de la enfermedad por casos y la importancia de los profesionales de la salud en la educação para la salud y el diagnóstico adecuado de la enfermedad.

## INTRODUCTION

Leprosy is an infectious disease with a high potential for chronicity, transmitted by direct contact with droplets containing the bacillus *Mycobacterium leprae*, through the upper airways of an untreated sick individual. It is a disease of low pathogenicity, due to its slow pattern in the process of bacterial multiplication, requiring intimate and prolonged contact for the development of clinical manifestations, with an incubation period of on average two to five years^(1-3)^.

Leprosy is considered a public health concern in several countries, due to its high prevalence and incidence rates^(1,4)^. In 2021, the World Health Organization reported 140,594 new cases of leprosy in 106 countries. The detection rate of new cases increased by 10.2% compared to the previous year. In the Americas, there were 19,826 (14.1%) reported cases; of these, 18,318 (92.4%) occurred in Brazil, ranking second among countries with the highest number of cases in the world^(5,6)^.

Among the challenges in the diagnosis and polychemotherapy (PCT) treatment of people with leprosy, the literature has been unanimous in reporting treatment abandonment as an obstacle to eradication of leprosy worldwide, as inadequate treatment does not establish a cure and, consequently, transmission is not broken, leading to a continuous cycle of leprosy^(1,2,4,7-10)^. Other implications include a greater risk of developing disabilities, risk of antimicrobial resistance to antibiotics, deformities and, consequently, suffering from prejudices and social stigmas^(7,8)^.

The treatment regimen considered inappropriate depends on the operational form of the pathology manifested. Patients with paucibacillary (PB) who have not completed the recommended treatment within the equivalent period of up to nine months are not discharged as cured and must restart PCT. Similarly, patients with multibacillary (MB) disease must complete treatment within the corresponding period of up to 18 months; after this time, it is considered ineffective and must restart PCT from the beginning^(2,9)^.

In this regard, this study is dedicated to analyzing the problem of non-adherence to drug treatment in leprosy, considering the endemicity of the northern region of the country and the implications of abandoning PCT treatment. It is expected that understanding factors that influence abandonment will contribute to the development of effective strategies and policies for treatment and, consequently, the reduction of incidence of leprosy.

## OBJECTIVES

To identify factors associated with non-adherence to PCT treatment for leprosy in a capital city in the north of the country.

## METHODS

### Ethical aspects

Before starting, this study was assessed and approved by the *Fundação Escola de Saúde Pública* (FESP) Research Project Approval Committee, which regulates research projects in the municipal network, and by the FESP Research Ethics Committee. Research participants were previously informed about the objectives, risks and benefits of the research, as well as their freedom to participate and the responsibility for confidentiality of researchers. Data collection began only after the clarifications, acceptance of research participants and signing of the Informed Consent Form.

### Study design, period and place

This is a cross-sectional observational study, which was guided by the Strengthening the Reporting of Observational studies in Epidemiology tool. The study was carried out in the city of Palmas, state of Tocantins, the youngest capital in the country, created in 1988, and currently with a population of 302,692 people^(11)^. The municipality belongs to the North region, and its main biome is the *cerrado* (vast ecoregion of tropical savanna in central Brazil), geographically located in the center of Brazil. Its healthcare network during the study period was composed of 34 Basic Health Units (BHUs), six rural healthcare points, 86 Family Health teams (which corresponds to 100% coverage), 75 Oral Health teams and 15 Family Health Support Centers, distributed across eight health territories in the municipality.

During the collection period, care for people with leprosy took place mainly within the scope of primary care, being carried out mainly by physicians and nurses, using the care protocol recommended by the Ministry of Health^(2)^. Other professionals such as physiotherapists and dentists also shared the care of people with leprosy. The study was conducted from 2019 to 2020, with the data collection period being in the second half of 2019.

### Population, inclusion and exclusion criteria

The study included all cases of leprosy diagnosed in BHUs, over 18 years old, residents of the city of Palmas and who had a history of treatment abandonment in the proposed period. Duplicate cases in the Notifiable Diseases Information System (In Portuguese, *Sistema de Informação de Agravos de Notificação* - SINAN) were excluded as well as those not found in the registry of BHUs or those that were not located after three attempts to search homes carried out with health workers. The criteria used to classify cases of abandonment of drug treatment for leprosy were those established by the Ministry of Health^(3)^: 1) MB â‰¥ six months late; and 2) PB â‰¥ three months late.

### Study protocol

For the initial selection of the study population, data from the Ministry of Health's SINAN, authorized and made available by the Municipal Health Department, corresponding to the period from January 2017 to December 2018, were used.

In the initial phase of this study, cases of leprosy treatment abandonment were identified and selected from SINAN, using the variable "TP_ALTA" to filter the data, which refers to the type of discharge received by patients, selecting "7 : abandonment" and the variable "DT_NOTIFIC", referring to the date of notification of the case, selecting the cases notified in the years 2017 and 2018. In the next phase, coordination with BHU teams took place: meetings with the coordinators of each unit and explanation of the objectives and operationality of the research; meetings with health workers, seeking assistance in communication and searching for users who have abandoned treatment. Data collection began shortly thereafter by applying a structured questionnaire containing 30 questions related to sociodemographic (gender, age, race/skin color, health territory, education, occupation, marital status, religion) and socioeconomic (*per capita* income, housing conditions) aspects, in addition to lifestyle habits (smoking and alcohol use), comorbidities (hypertension, diabetes, mental illness, dyslipidemia, obesity), access to healthcare services, guidance and questions about leprosy and treatment, and motivation for abandoning leprosy treatment. The questionnaire was constructed and applied by the authors, and a pilot study with five people was previously conducted to adapt the questionnaire. However, as there was no change in the collection instrument, these people were included in the sample.

The patients included in the study were contacted via a health worker and/or telephone, and the conversation with the researcher was scheduled to take place at the reference BHU or at participants' home, according to research participants' preference. When carried out at home, the visit was always accompanied by health workers. The questionnaire was applied in a reserved room and/or place, restricted to the researcher and participant, regardless of the location (BHU or home), to guarantee participant privacy and confidentiality.

### Analysis of results, and statistics

The data were tabulated using Microsoft Excel^®^, and Stata 17^®^ was used for analysis. The data were presented in aggregate form, using simple frequencies and percentages to verify the prevalence of each nominal variable in the outcome. For bivariate analyses of categorical variables, nonparametric tests were performed, such as X^2^ and Fisher's exact test (when recommended), and for continuous variables (age), the Mann-Whitney U test was performed, after verifying nonnormal distribution by the Shapiro-Wilk test.

The Poisson regression model with robust variance estimator was used for multivariate analysis. We used this model because the odds ratio estimated by cross-sectional logistic regression may significantly overestimate the relative risk when the outcome is common^(12,13)^. Second, in case of convergence failure with the log-binomial model, Poisson regression with a robust variance performs better in estimating the prevalence ratio (PR) of a cross-sectional study^(14)^.

The independent variables that were significant in bivariate analysis (p<0.20) were inserted into the initial multivariate model. After that, the variables that presented the highest p-value were removed one by one, leaving only those considered statistically significant (p<0.05) in the final model. The strength of association was calculated using the PR and 95% confidence interval (95%CI). The adequacy of models was assessed according to the pseudo R^2^ value (used for binary outcomes), which showed how much the model can explain the variation in the data presented: the higher the pseudo R^2^, the better the model. Moreover, the Akaike Information Criterion and the Bayesian Information Criterion were used, in which the lowest value is expected to provide the best fit.

## RESULTS

During the study period, 2017 and 2018, the overall detection rate of new cases in Palmas was 183.76/100,000 and 271.37/100,000, respectively. According to selection criteria, in 2017, 59 cases of treatment abandonment were identified, and in 2018, 29 cases, totaling 88 cases of abandonment. Abandonment rates for 2017 and 2018 were 8.3% and 3.12%, respectively. Of the 88 cases of abandonment identified in the system, 43 were not located by telephone or by the health team; four were minors; and one did not agree to participate in the research, totaling a final sample of 40 patients who participated in this study.

The results of the sociodemographic characteristics collected from research participants are available in Table 1. Among the study participants, there is a predominance of women (57.5%), with an average age of 48.2 years, ranging from 20 to 83 years. In addition, 75% declared themselves black or brown, with an average *per capita* income of 608 *reais*, 50% with complete or incomplete elementary education, 52.5% retired or unemployed and 60% without a partner.

**Table 1:** Frequency distribution of sociodemographic characteristics and association with non-adherence to drug treatment for leprosy in the period 2017-2018, Palmas, Tocantins, Brazil (N=40)

Variables	n(%)	Still not returning to treatment		*p* value
		Yes (%) (n=23)	No (%) (n=17)	
Health territory				
Karajá	8(20)	5(62.5)	3(37.5)	0.762^a^
Xambioá	5(12.5)	2(40)	3(60)	
Kanela	9(22.5)	6(66.7)	3(33.3)	
Xerente	11(27.5)	7(63.6)	4(36.4)	
Javaé	3(7.5)	1(33.3)	2(66.7)	
Krahô	3(7.5)	1(33.3)	2(66.7)	
Apinajé	1(2.5)	1(100)	0(0)	
Sex								
Male	17(42.5)	6(35.3)	11(64.7)	**0.015** ^a^
Female	23(57.5)	17(74)	6(26)	
Age range				
20 : 30 years	6(15)	4(67)	2(33)	0.166^a^
31 : 50 years	19(47.5)	8(42.1)	11(57.9)	
51 : 83 years	15(37.5)	11(73.3)	4(26.7)	
Ethnicity/skin color				
Black/brown	30(75)	16(53.3)	14(46.7)	0.471^b^
White/yellow/indigenous	10(25)	7(70)	3(30)	
Marital status				
Without a partner	16(40)	8(50)	8(50)	0.433^a^
With a partner	24(60)	15(62.5)	9(37.5)	
Education				
Up to complete elementary education	24(60)	13(54.2)	11(45.8)	0.601^a^
Complete secondary school to higher education	16(40)	10(62.5)	6(37.5)	
Religion				
Catholic/Evangelical	31(77.5)	16(51.6)	15(48.4)	0.256^b^
No	9(22.5)	7(77.8)	2(22.2)	
Occupation				
Self-employed/administrative	19(47.5)	8(42.1)	11(57.9)	0.061^a^
Retired/unemployed	21(52.5)	15(71.4)	6(28.6)	
*Per capita* income	R$608.33^c^	R$554.87	R$680.66	0.277^a^
Housing				
Own	29(72.5)	16(55.2)	13(44.8)	0.730^b^
Rented/other	11(27.5)	7(63.6)	4(36.4)	
Zone				
Urban	38(95)	22(57.9)	16(42.1)	1.000^b^
Rural	2(5)	1(50)	1(50)	
Asphalt street				
Yes	33(82.5)	19(57.6)	14(42.4)	1.000^b^
No	7(17.5)	4(57.2)	3(42.8)	
Treated water				
Yes	39(97.5)	23(59)	16(41)	0.425^b^
No	1(2.5)	0	1(100)	
Treated sewage				
Yes	24(60)	14(58.3)	10(41.7)	0.896^a^
No	16(40)	9(56.3)	7(43.7)	
Street lighting				
Yes	38(95)	22(57.9)	16(42.1)	1.000^b^
No	2(5)	1(50)	1(50)	
Public transport				
Yes	38(95)	22(57.9)	16(42.1)	1.000^b^
No	2(5)	1(50)	1(50)	

a Pearson's chi-square test;.

b Fisher's exact test;.

c average; n - total number of patients included; per capita income = family income/number of people in the household in reais.

The results of health characteristics, lifestyle habits, access to healthcare services and reasons for abandoning leprosy treatment collected from participants are presented in Table 2. It was found that, among people with leprosy who abandoned treatment, 42.5% had already resumed therapy, 42.5% intended to resume and 15% did not intend to. In view of this, an association analysis was performed between the other variables and the outcome "still not returning to treatment". Bivariate analysis showed that the following were risk factors for continuing without treatment: being male, having doubts about treatment and/or disease, having diabetes, not having improvement in symptoms after starting treatment, and having another physician rule out leprosy diagnosis/not believing that one has leprosy.

**Table 2:** Frequency distribution of health characteristics, lifestyle habits, access to healthcare services and reasons for abandoning leprosy treatment and association with non-adherence to drug treatment for leprosy in the period 2017-2018, Palmas, Tocantins, Brazil (N=40)

Variables	n(%)	Still not returning to treatment		*p* value
		Yes (%) (n=23)	No (%) (n=17)	
Smoking				
Yes	7(17.5)	3(42.8)	4(57.2)	1.000^b^
No	33(82.5)	19(57.6)	14(42.4)	
Alcohol use				
Yes	24(60)	13(54.2)	11(45.8)	0.601^a^
No	16(40)	10(62.5)	6(37.5)	
High blood pressure				
Yes	13(32.5)	3(23)	10(77)	0.103^b^
No	27(67.5)	14(51.8)	13(48.2)	
Diabetes				
Yes	6(15)	6(100)	0(0)	**0.030** ^b^
No	34(85)	17(50)	17(50)	
Obesity				
Yes	2(5)	2(100)	0(0)	0.499^b^
No	38(95)	21(55.3)	17(44.7)	
Difficulty accessing healthcare services?				
Yes	5(12.5)	2(40)	3(60)	0.634^b^
No	35(87.5)	21(60)	14(40)	
Did you receive guidance about illness/treatment?				
Yes	38(95)	21(55.3)	17(44.7)	0.499^b^
No	2(5)	0(0)	2(100)	
Do you have any questions about leprosy/treatment?				
Yes	17(47.5)	2(11.7)	15(88.3)	**0.001** ^b^
No	23(57.5)	15(65.2)	8(34.8)	
Did symptoms improve after starting treatment?				
Yes	24(60)	8(33.3)	16(66.7)	**0.000** ^b^
No	16(40)	15(93.7)	1(6.3)	
Drug reaction?				
Yes	27(67.5)	11(40.7)	16(59.3)	0.746^a^
No	13(32.5)	6(46.2)	7(53.8)	
Did you receive a visit from a community health worker?				
Yes	38(95)	21(55.3)	17(44.7)	0.212^b^
No	2(5)	2(100)	0(0)	
Did you report the illness to others?				
Yes	33(82.5)	20(60.6)	13(39.4)	0.432^b^
No	7(17.5)	3(42.8)	4(57.2)	
Did you feel discriminated against?				
Yes	21(52.5)	12(57.2)	9(42.8)	0.962^a^
No	19 (47.5)	11(57.9)	8(42.1)	
Why did you give up on treatment?				
Another physician ruled out the diagnosis/does not believe he has leprosy	20(50)	14(73.7)	5(26.3)	**0.049** ^a^
Medication reaction/alcohol use/other reasons	20(50)	9(42.8)	12(57.2)	

a Pearson's chi-square test;.

b Fisher's exact test; n - total number of patients included.

The multivariate analysis model demonstrated that the variables "no improvement in signs and symptoms" and "having leprosy diagnosis ruled out by another physician" and/or "not believing in leprosy" were significant factors in continuing without leprosy treatment (Table 3).

**Table 3:** Robust Poisson regression model to estimate prevalence ratios of factors associated with continued non-treatment of leprosy in the period 2017-2018, Palmas, Tocantins, Brazil (N=40)

Variables	Initial model		*p* value	Final model		*p* value
	PR (95%CI)	PR (95%CI)				
Sex				
Male	0.94(0.53-1.67)	0.856	-	-
Female	1		-	-
Occupation				
Self-employed/administrative	1		-	-
Retired/unemployed	1.70(1.05-2.75)	0.028	-	-
Diabetic				
No	1		-	-
Yes	1.13(0.74-1.73)	0.551	-	-
Question about leprosy/treatment				
No	1		1	
Yes	1.84(1.10-3.08)	0.020	1.93(1.10-3.39)	0.02
Improvement of symptoms				
Yes	1		1	
No	1.92(1.14-3.23)	0.013	2.25(1.26-4.04)	0.00
Reason for discontinuing treatment				
Another physician ruled out the diagnosis/does not believe he has leprosy	1.44(0.88-2.36)	0.141	-	-
Medication reaction/alcohol use/other reasons	1		-	-
Constant	0.11(0.03-0.38)	0.000	0.26(0.14-0.47)	0.000
Pseudo R^2^	13.6%		11.3%	
Akaike Information Criterion	75.733		69.374	
Bayesian Information Criterion	87.555		74.441	

PR - prevalence ratio; 95%CI - 95% confidence interval.

Table 4 shows that 37.5% of participants reported the need for service improvements, with customer service/reception being the most mentioned sector (25%). Among the 23 participants who still have not returned to treatment, only six people reported an intention to resume, with the main impediment cited for this return being the report of considering themselves cured and no longer having leprosy (42.5%).

**Table 4:** Characteristics of the healthcare service and non-adherence of patients identified by the Notifiable Diseases Information System as cases of abandonment of leprosy treatment in the period 2017-2018, Palmas, Tocantins, Brazil (N=40)

Characteristics	n(40)	%
Do you identify the need to improve the service?		
Yes	15	37.5
No	25	62.5
What needs to be improved in the healthcare service?		
Reception/service	10	25
More medical professionals	5	12.5
Not applicable	25	62.5
Do you intend to resume treatment?		
Yes	17	42.5
No	6	15
Already returned	17	42.5
What is your impediment to resuming treatment?		
Reports not having a disease or being cured	17	42.5
Others (alcohol use, reaction to medication, difficulty accessing)	6	15
Not applicable	17	42.5

n - total number of patients included; % - percentage.

## DISCUSSION

Leprosy has high incidence rates in the north region of the country, specifically in the state of Tocantins, and is considered a hyperendemic region with active and continuous transmission^(15,16)^. Brazilian regions with the highest leprosy burdens (North, Northeast and Central-West regions of Brazil) have greater chances of abandoning treatment in relation to regions such as the South and Southeast, with a lower burden^(7)^.

In this context, abandonment of leprosy treatment represents one of the most relevant challenges to the control of chronic infectious diseases that require long-term treatment^(17)^. A study using mathematical modeling demonstrated that non-adherence to PCT and abandonment can have a negative impact on eradication of leprosy, leading to an increase in the prevalence of leprosy and related deaths worldwide^(18)^.

Our study shows dropout rates of 8.3% and 3.12% in 2017 and 2018, respectively, higher than those found in other studies^(7,10,17)^. The results of this study indicate that the lack of improvement in symptoms and diagnosis dismissal by another physician as well as the lack of belief in the presence of leprosy are barriers to adherence to leprosy treatment. Furthermore, bivariate analysis revealed that male gender, the presence of diabetes and doubts about treatment are associated with non-return to PCT.

There is a lack of published studies investigating factors associated with abandonment of leprosy treatment^(7,8,10,17,19)^, and most do not explore characteristics such as reasons for abandonment, access to and quality of healthcare services, information about leprosy, doubts and discrimination, as was done in this study. The challenge of carrying out research such as this is highlighted, given the difficulties in searching for and finding cases of abandonment, as mentioned in other studies^(8)^.

Improvement in leprosy symptoms is already known as a condition that favors abandonment of leprosy treatment^(7,8)^. However, although 60% of participants reported improvement in symptoms after using PCT, our results demonstrate that the lack of improvement in symptoms is related to non-return to treatment. This result suggests the need for more robust studies in this field that address qualitative factors related to treatment abandonment by leprosy patients, such as acceptance of leprosy, and aspects of psychological, social and cultural domains.

In 73.7% of cases of abandonment that remained untreated, the reason for abandonment was diagnosis dismissal by another physician (usually from the private network) or the lack of belief in the presence of leprosy. The lack of studies that demonstrate such a result demands reflection as well as the carrying out of future research to investigate this issue in greater depth.

Qualitative research on abandonment of leprosy treatment highlights the fragility of patient reliability regarding medical diagnosis and the lack of acceptance of the use of PCT as a tool for achieving a cure for leprosy^(20,21)^. It is known that leprosy is a pathology that carries a lot of stigma. Thus, many patients, because they have negative feelings about leprosy, end up not accepting an initial diagnosis, opting to seek another professional's opinion, but this professional, in turn, may be somewhat unprepared and dismiss diagnosis^(22)^. People suspected of having leprosy end up going through a long journey in search of an answer about their health condition, and often receive a mistaken diagnosis that can lead to serious future consequences^(23)^.

Even though they are aware of the pathology, some people affected may reject the idea of having leprosy and report not feeling any type of symptom^(21)^. It is believed that the intensity of the feeling of non-acceptance by patients may make the process of adherence to treatment even more difficult, especially in patients who do not present symptoms characteristic of leprosy, increasing dissatisfaction with diagnosis due to internalized prejudiced concepts. All psychological or social problems caused by leprosy are inherent to the prejudice, discrimination and stigma existing in individuals and in society^(24)^.

It is clear that the manifestation of denial of leprosy is a condition present in most patients, as it is still a stigmatized disease. Denial is characterized by a defense mechanism in which reality is denied as a way of avoiding suffering, whether physical or psychological. In this regard, it is extremely important for the team of professionals to consider these manifestations from the diagnosis and to monitor patients, since adherence to treatment, changes in lifestyle and behavior are based on the principle that patients accept their condition and become a co-responsible worker for the recovery of their health^(20-22)^.

There was a predominance of males in 61.25% of cases of abandonment in 2017 and 2018. However, after the active search for research collection, the sample consisted of a majority of females (57.5%), demonstrating the difficulty in reaching and identifying male individuals in a situation of abandonment of leprosy treatment. Studies indicate that men are more resistant to seeking healthcare services^(8,10)^, resulting in less guidance on leprosy and interfering with adherence to and follow-up of treatment^(25)^.

Regarding color/ethnicity, it is known that leprosy can affect any individual. However, health indicators in Brazil, based on this variable, have shown that black and mixed-race people have higher numbers when compared to the white population^(7)^. This fact may be the result of social inequalities that still exist in the country, requiring more related studies to understand the distribution in this group and associated factors^(7,26)^. It is worth noting that the state of Tocantins has a higher proportion of black and brown people compared to other categories^(11)^.

The results show that 60% of participants had completed elementary school, of which 10% were illiterate, evidencing a low level of education among dropout cases, as demonstrated in other studies^(7,10)^. Findings that may constitute limitations in the process of understanding the pathology and its treatment by patients, triggering greater resistance to treatment adherence, adequate follow-up and episodes of abandonment of PCT.

Alcohol use was reported by 60% of participants in this study, of which 15% reported consuming alcoholic beverages daily, characterizing dependence. In addition to being a global problem that influences behavior and harms the health of those affected, alcohol consumption is a factor that negatively interferes in the process of accepting leprosy and in the use of medication. It can also reduce the healing effect of medications and be a predictive factor for disease recurrence^(10,27)^.

The correlation between alcohol and treatment abandonment is already recognized^(7,28)^, as alcohol can lead to forgetting to take daily medications or be contraindicated during medical treatments. Consequently, some people may choose to continue consuming alcohol, even to the detriment of therapy, either due to an unfavorable social and educational level (which makes it difficult to recognize the importance of treatment) or chemical dependency^(7)^. In this context, there is a need for a multidisciplinary and intersectoral approach to the management and monitoring of these patients, especially the support of the specialized care network for patients who abuse alcohol and drugs.

The presence of comorbidities in people with leprosy can increase the risk of not following drug treatment correctly, as the concomitant use of several daily medications can leave patients confused about their use, cause discomfort due to drug interactions, making them more vulnerable to abandonment^(10,29)^. In this study, 100% of people with diabetes and leprosy persisted without returning to leprosy treatment.

It is understood that at the time of diagnosis of any disease, the team of professionals must offer support to patients and provide appropriate guidance regarding leprosy process and its treatment so that patients have no doubts and can thus be leading figures in the reestablishment of their health. Knowing the proportional relationship between information and abandonment of treatment^(7)^, in this study 95% reported receiving guidance about leprosy and its treatment at the time of diagnosis, but 42.5% reported still having doubts about leprosy as well as its treatment. This variable was significantly associated with non-return to leprosy treatment in bivariate analysis. Thus, some questions can be raised regarding the health education processes carried out by the city's health teams, mainly related to the qualification of health team members, patients' prior knowledge about leprosy, the inclusion of family in treatment, among others.

This weakness may be associated with the lack of knowledge of many professionals about leprosy, treatment and its adverse effects, thus limiting the health education process, or it may be related to guidance given in inappropriate language, not considering patients' level of understanding^(23)^. In view of this, it highlights the importance of ongoing education actions to improve quality of care in leprosy clinical management. It is also necessary to establish a therapeutic relationship between professionals and patients, working on aspects of empathy and holisticity during consultations, especially at the time of diagnosis, considering prior knowledge, education, beliefs, fears and insecurities, helping in a better understanding and adherence to treatment^(30)^.

In this study, 52.5% reported discrimination at some point after diagnosis. Leprosy is a disease full of distortions, stigmas, and prejudices that directly affect adherence to treatment, causing conflicts within the family and society, in addition to causing psychological distress to patients, interfering with daily activities, work, and quality of life. In more severe cases, it leads to loss of self-esteem and depression, where primary healthcare services are often unable to provide satisfactory care, leading these people to isolation. Thus, there is a need to improve professionals' skills and perceptions regarding leprosy comprehensive and quality management^(31)^.

It is important to highlight the weaknesses in health teams' work processes in assisting people with leprosy, such as the "loss" of cases in the health network, which results in discontinuity of care. This study shows that, even with active search, it was not possible to identify the location of 53% of leprosy abandonment cases. This scenario highlights the importance of primary care as a care organizer in the network, monitoring the therapeutic trajectory of these people within the network, acting in the active search for abandonment cases.

Likewise, cases of abandonment in leprosy treatment point to the importance of monitoring through home visits by community health workers, teamwork and guidance on permanent sequels and practice-oriented training of professionals, among others^(32)^.

Considering the adverse effects caused by PCT, which can cause daily physical discomfort to users, often associated with resistance to treatment adherence and, in many cases, abandonment. This study shows that 25% of patients participating in the study abandoned leprosy treatment due to reactions to medications. The Ministry of Health, aiming to combat adverse reactions and drug intolerance developed during treatment, established protocols for replacing regimens, aiming to eliminate the undesirable effects of PCT^(26)^. Antimicrobial resistance is also evident, frequently reported as a consequence of episodes of therapeutic abandonment^(25)^. Thus, the role of healthcare professionals is highlighted in monitoring patients undergoing treatment in the face of side effects, seeking to offer support and assessing the need to establish another recommended alternative scheme, with the aim of conducting a treatment with fewer undesirable effects, promoting a better quality of life while following the therapeutic regimen.

### Study limitations

It is important to recognize the limitations inherent to this research, such as the number of cases of treatment abandonment due to operational difficulties, such as user location (either due to change of address or incomplete address in SINAN and in medical records). In addition, due to the cross-sectional nature of this study, it was not possible to establish causal factors, as well as the uniqueness of the location studied, which may not reflect the reality of other locations in the country. In this sense, we recommend conducting further research using methodologies that allow identifying other possible factors related to non-adherence to leprosy treatment.

### Contributions to nursing, health or public policy

The study contributes to the identification of factors involved in the abandonment of PCT treatment for leprosy in a hyper-endemic capital in the north of the country, highlighting variables not previously described, such as the lack of improvement in symptoms, having the diagnosis ruled out by another physician and not believing they have leprosy, as the most relevant.

## CONCLUSIONS

Factors associated with non-adherence to PCT treatment for leprosy, identified by bivariate analysis in this study, were male gender, doubts about treatment and/or leprosy, presence of diabetes, lack of improvement in symptoms after starting treatment as well as the fact that another physician ruled out leprosy diagnosis or the person did not believe they had leprosy. After multivariate analysis, it was observed that the lack of improvement in signs and symptoms and leprosy diagnosis ruled out by another physician, or the lack of belief in leprosy, were significant for the model. The study reveals important findings related to non-adherence to leprosy treatment, providing support for the implementation of actions and strategies that improve quality of care, in addition to guiding future research that aims to understand in more depth the intrinsic factors as well as those related to the service and care provided to people with leprosy.

## Data Availability

The research data are available within the article.
