# Supplementary material for: Factors associated with non-adherence to polychemotherapy treatment for leprosy
Source: Rev Bras Enferm. 2025 Dec 8;78(4):e20240437. doi: 10.1590/0034-7167-2024-0437 (PMC12716807; doi:10.1590/0034-7167-2024-0437)
Supplement: Translation

# Translation: Fatores associados à não adesão ao tratamento poliquimioterápico para hanseníase

**DOI:** 10.1590/0034-7167-2024-0437pt

**Authors:** Johnne Silva da Cruz, Eliane Patricia Lino Pereira Franchi

**Affiliations:** I Fundação Escola de Saúde Pública de Palmas, Palmas, Tocantins, Brasil.

**Keywords:** Hanseníase, Fatores Desencadeantes, Não Adesão à Medicação, Recusa do Paciente ao Tratamento, Atenação Primária à Saúde

## Abstract

**Objetivos:**

identificar os fatores associados à não adesão ao tratamento poliquimioterápico para hanseníase.

**Métodos:**

estudo transversal, realizado com casos de abandono do tratamento para hanseníase no município de Palmas-TO. Os casos foram identificados a partir do Sistema de Notificação de Agravos de Notificação, sendo, posteriormente, aplicado um questionário.

**Resultados:**

as taxas de abandono para os anos de 2017 e 2018 foram de 8,3% e 3,12%, respectivamente. Quarenta pessoas participaram do estudo: maioria mulheres (57,5%), média de idade de 48,2 anos, com 75% de pretos/pardos, 50% com ensino fundamental completo/incompleto e 52,5% aposentados/desempregados. Os fatores associados à adesão ao tratamento da hanseníase foram a não melhora dos sintomas e ter o diagnóstico descartado por outro médico/não acreditar ter a doença.

**Conclusões:**

os resultados apontam para a dificuldade de aceitação da doença pelos casos e a importância dos profissionais de saúde na educação em saúde e diagnóstico adequado da doença.

## INTRODUÇÃO

A hanseníase é uma doença infectocontagiosa com alto poder de cronicidade, transmitida pelo contato direto com gotículas contendo o bacilo *Mycobacterium leprae*, através das vias aéreas superiores do indivíduo doente não tratado. É uma doença de baixa patogenicidade, devido apresentar um padrão lento no processo de multiplicação bacteriana, necessitando de contato íntimo e prolongado para o desenvolvimento das manifestações clínicas, com período de incubação compreendendo em média dois a cinco anos^(1-3)^.

A hanseníase é considerada um problema de saúde pública em vários países, mediante os altos índices de prevalência e incidência^(1,4)^. Em 2021, a Organização Mundial da Saúde reportou 140.594 casos novos da doença em 106 países. A taxa de detecação de casos novos elevou 10,2% em comparação com ano anterior. Nas Américas, houve 19.826 (14,1%) casos notificados; desses, 18.318 (92,4%) ocorreram no Brasil, ocupando o segundo lugar entre os países com maior número de casos no mundo^(5,6)^.

Entre os desafios no diagnóstico e tratamento poliquimioterápico (PQT) de pessoas com hanseníase, a literatura tem sido uníssona em relatar o abandono do tratamento como um entrave na erradicação da doença em à ¢mbito mundial, pois o tratamento inadequado não estabelece a cura e, consequentemente, a transmissão não é rompida, levando a um ciclo contínuo da hanseníase^(1,2,4,7-10)^. Outras implicações residem no maior risco no desenvolvimento de incapacidades, risco de resistência antimicrobiana aos antibióticos, deformidades e, consequentemente, o sofrimento de preconceitos e estigmas sociais^(7,8)^.

O regime de tratamento considerado inapropriado depende da forma operacional da patologia manifestada. Acometidos paucibacilares (PB) que não concluíram o tratamento preconizado no período equivalente até nove meses não recebem alta por cura e precisam reiniciar a PQT. Da mesma maneira, os multibacilares (MB) devem concluir o tratamento no período correspondente até 18 meses; acima deste tempo, é considerado ineficaz, necessitando retomar a PQT do início^(2,9)^.

Neste sentido, o presente estudo dedica-se à análise da problemática da não adesão ao tratamento medicamentoso na hanseníase, considerando a endemicidade da região norte do país e as implicações do abandono do tratamento PQT. Espera-se que a compreensão dos fatores que influenciam o abandono contribua para a elaboração de estratégias e políticas eficazes para o tratamento e, consequentemente, a diminuiação da incidência da doença.

## OBJETIVOS

Identificar fatores associados à não adesão ao tratamento PQT para hanseníase em uma capital do norte do país.

## MÉTODOS

### Aspectos éticos

Antes do início, este estudo recebeu apreciação e aprovação da Comissão de Aprovação de Projetos de Pesquisa da Fundação Escola de Saúde Pública (FESP), a qual regula projetos de pesquisa na rede municipal e do Comitê de Ética em Pesquisa da FESP. Os participantes da pesquisa foram, previamente, esclarecidos sobre os objetivos, riscos e benefícios da pesquisa, assim como liberdade de participação e responsabilidade de sigilo dos pesquisadores. Apenas após esclarecimentos, aceite dos participantes da pesquisa e assinatura do Termo de Consentimento Livre e Esclarecido, iniciou-se a coleta de dados.

### Desenho, período e local do estudo

Trata-se de estudo observacional transversal, que foi norteado pela ferramenta *Strengthening the Reporting of Observational studies in Epidemiology*. O estudo foi realizado na cidade de Palmas, estado do Tocantins, mais jovem capital do país, sendo criada em 1988, atualmente com população de 302.692 pessoas^(11)^. O município pertence à região Norte, e seu principal bioma é o cerrado, localizado geograficamente no centro do Brasil. Sua rede assistencial no período de estudo estava composta por 34 Unidades Básicas de Saúde (UBSs), seis pontos de atenação à saúde rural, 86 equipes de Saúde da Família (o que corresponde a 100% de cobertura), 75 equipes de Saúde Bucal e 15 Núcleos de Apoio à Saúde da Família, distribuídos em oito territórios de saúde no município.

No período de coleta, os atendimentos à s pessoas com hanseníase aconteceram, majoritariamente, no à ¢mbito da atenação primária, sendo realizados, principalmente, por médicos e enfermeiros, utilizando-se o protocolo assistencial preconizado pelo Ministério da Saúde^(2)^. Outros profissionais como fisioterapeutas e odontólogos também compartilhavam o cuidado à s pessoas com hanseníase. A realização do estudo ocorreu de 2019 a 2020, sendo o período de coleta executado no segundo semestre de 2019.

### População, critérios de inclusão e exclusão

Foram incluídos no estudo todos os casos de hanseníase diagnosticados nas UBSs, maiores de 18 anos, moradores do município de Palmas e que apresentaram histórico de abandono do tratamento no período proposto. Foram excluídos os casos duplicados no Sistema de Informação de Agravos de Notificação (SINAN), assim como aqueles não encontrados no cadastro das UBSs ou os que não foram localizados após três tentativas de busca domiciliar realizadas junto aos agentes de saúde. Os critérios utilizados para classificar os casos de abandono de tratamento medicamentoso de hanseníase foram os estabelecidos pelo Ministério da Saúde^(3)^: 1) MB â‰¥ seis meses de atraso; e 2) PB â‰¥ três meses de atraso.

### Protocolo do estudo

Para a seleação inicial da população do estudo, foram utilizados os dados do SINAN do Ministério da Saúde, autorizados e disponibilizados pela Secretaria Municipal de Saúde, correspondentes ao período de janeiro de 2017 a dezembro de 2018.

Na fase inicial do estudo, os casos de abandono de tratamento de hanseníase foram identificados e selecionados a partir do SINAN, utilizando-se na filtragem dos dados a variável "TP_ALTA", que remete ao tipo de alta recebida pelos pacientes, sendo selecionados "7 : abandono" e a variável "DT_NOTIFIC", referente à data de notificação do caso, selecionando os casos notificados nos anos de 2017 e 2018. Na fase seguinte, ocorreu a articulação com as equipes das UBSs: reuniões com os coordenadores de cada unidade e explanação dos objetivos e a operacionalidade da pesquisa; reuniões com os agentes de saúde, buscando auxílio na comunicação e procura pelos usuários em abandono. Logo após, iniciou-se a coleta de dados com a aplicação de questionário estruturado contendo 30 questões relacionadas aos aspectos sociodemográficos (gênero, idade, raça/cor da pele, território de saúde, escolaridade, ocupação, situação conjugal, religião) e socioeconômicos (renda *per capita*, condições de moradia), além de hábitos de vida (tabagismo e uso de álcool), comorbidades (hipertensão, diabetes, doença mental, dislipidemia, obesidade), acesso a serviços de saúde, orientações e dúvidas sobre doença e tratamento, e motivação para o abandono do tratamento da hanseníase. O questionário foi construído e aplicado pelos autores, e estudo piloto com cinco pessoas foi conduzido previamente para a adequação do questionário. Contudo, como não houve alteração no instrumento de coleta, essas pessoas foram incluídas na amostra.

Os pacientes incluídos no estudo foram contatados via agente de saúde e/ou telefone, e a conversa com o pesquisador foi agendada para ocorrer na UBS de referência ou no domicílio, conforme preferência do participante da pesquisa. Quando realizada em domicílio, a visita sempre foi acompanhada pelo agente de saúde. A aplicação do questionário, independentemente do local (UBS ou domicílio), ocorreu em sala e/ou local reservado, restrito a pesquisador e participante, para garantia de privacidade e sigilo do participante.

### Análise dos resultados e estatística

Os dados foram tabulados utilizando o *software Microsoft Excel^®^*, e para análise, foi utilizado o Stata 17^®^. Os dados foram apresentados de forma agregada, utilizando-se frequências simples e porcentual para verificar a prevalência de cada variável nominal no desfecho. Para análises bivariadas de variáveis categóricas, realizaram-se testes não paramétricos, como X^2^ e teste exato de Fisher (quando recomendável), e para variável contínua (idade), realizou-se o teste U de Mann-Whitney, após verificar distribuiação não normal pelo teste de Shapiro-Wilk.

O modelo de regressão de Poisson com estimador de variância robusta foi utilizado para análise multivariada. Usamos esse modelo porque a razão de chances estimada por meio de regressão logística de estudo transversal pode superestimar significativamente o risco relativo quando o desfecho é comum^(12,13)^. Em segundo lugar, no caso de falha de convergência com o modelo *log-binomial*, a regressão de Poisson com uma variância robusta tem melhor desempenho na estimativa da razão de prevalência (RP) de estudo transversal^(14)^.

No modelo multivariado inicial, inseriram-se as variáveis independentes que se apresentaram significativas na análise bivariada (p<0,20). Após isso, removeram-se uma a uma as variáveis que apresentavam o maior valor de p, permanecendo no modelo final apenas aquelas consideradas estatisticamente significantes (p<0,05). A força de associação foi calculada por meio da RP e intervalo de confiança de 95% (IC95%). A adequação dos modelos foi avaliada segundo o valor de pseudo R^2^ (utilizado para desfechos binários), que mostrou o quanto o modelo pode explicar a variação dos dados apresentados: espera-se que quanto maior o pseudo R^2^, melhor o modelo. Além disso, foram utilizados o Critério de Informação de Akaike e o Critério De Informação Bayesiano, em que se espera que o menor valor forneça o melhor ajuste.

## RESULTADOS

No período de estudo, 2017 e 2018, a taxa de detecação geral de casos novos em Palmas foi de 183,76/100 mil e 271,37/100 mil, respectivamente. Conforme critérios de seleação, no ano de 2017, foram identificados 59 casos de abandono de tratamento, e no ano de 2018, 29 casos, totalizando 88 casos de abandono. As taxas de abandono para o ano de 2017 e 2018 foram de 8,3% e 3,12%, respectivamente. Dos 88 casos de abandono identificados no sistema, 43 não foram localizados por telefone ou pela equipe de saúde; quatro eram menores de idade; e um não aceitou participar da pesquisa, totalizando-se uma amostra final de 40 pacientes que participaram deste estudo.

Os resultados das características sociodemográficas coletados dos participantes da pesquisa estão disponibilizados na Tabela 1. Entre os participantes do estudo, mostra-se uma predominância de mulheres (57,5%), com média de idade de 48,2 anos, variando de 20 a 83 anos. Além disso, 75% se autodeclararam pretos ou pardos, com média de renda *per capita* de 608 reais, sendo 50% com ensino fundamental completo ou incompleto, 52,5% aposentados ou desempregados e 60% não possuindo companheiro.

**Tabela 1:** Distribuiação de frequência das características sociodemográficas e associação com a não adesão ao tratamento medicamentoso para hanseníase no período de 2017-2018, Palmas, Tocantins, Brasil (N=40).

Variáveis	n(%)	Continua sem retornar ao tratamento		Valor de *p*
		Sim (%) (n=23)	Não (%) (n=17)	
Território de saúde				
Karajá	8(20)	5(62,5)	3(37,5)	0,762^a^
Xambioá	5(12,5)	2(40)	3(60)	
Kanela	9(22,5)	6(66,7)	3(33,3)	
Xerente	11(27,5)	7(63,6)	4(36,4)	
Javaé	3(7,5)	1(33,3)	2(66,7)	
Krahô	3(7,5)	1(33,3)	2(66,7)	
Apinajé	1(2,5)	1(100)	0(0)	
Sexo				
Masculino	17(42,5)	6(35,3)	11(64,7)	**0,015** ^a^
Feminino	23(57,5)	17(74)	6(26)	
Faixa etária				
20 : 30 anos	6(15)	4(67)	2(33)	0,166^a^
31 : 50 anos	19(47,5)	8(42,1)	11(57,9)	
51 : 83 anos	15(37,5)	11(73,3)	4(26,7)	
Etnia/cor da pele				
Preto/pardo	30(75)	16(53,3)	14(46,7)	0,471^b^
Branco/amarelo/indígena	10(25)	7(70)	3(30)	
Situação conjugal				
Sem companheiro	16(40)	8(50)	8(50)	0,433^a^
Com companheiro	24(60)	15(62,5)	9(37,5)	
Escolaridade				
Até fundamental completo	24(60)	13(54,2)	11(45,8)	0,601^a^
Ensino médio a superior completo	16(40)	10(62,5)	6(37,5)	
Religião				
Católica/evangélico	31(77,5)	16(51,6)	15(48,4)	0,256^b^
Não tem	9(22,5)	7(77,8)	2(22,2)	
Ocupação				
Autônomo/administrativo	19(47,5)	8(42,1)	11(57,9)	0,061^a^
Aposentado/desempregado	21(52,5)	15(71,4)	6(28,6)	
Renda *per capita*	R$608,33^c^	R$554,87	R$680,66	0,277^a^
Moradia				
Própria	29(72,5)	16(55,2)	13(44,8)	0,730^b^
Alugada/outro	11(27,5)	7(63,6)	4(36,4)	
Zona				
Urbana	38(95)	22(57,9)	16(42,1)	1,000^b^
Rural	2(5)	1(50)	1(50)	
Rua asfaltada				
Sim	33(82,5)	19(57,6)	14(42,4)	1,000^b^
Não	7(17,5)	4(57,2)	3(42,8)	
Água tratada				
Sim	39(97,5)	23(59)	16(41)	0,425^b^
Não	1(2,5)	0	1(100)	
Esgoto tratado				
Sim	24(60)	14(58,3)	10(41,7)	0,896^a^
Não	16(40)	9(56,3)	7(43,7)	
Iluminação na rua				
Sim	38(95)	22(57,9)	16(42,1)	1,000^b^
Não	2(5)	1(50)	1(50)	
Transporte coletivo				
Sim	38(95)	22(57,9)	16(42,1)	1,000^b^
Não	2(5)	1(50)	1(50)	

a Teste qui-quadrado de Pearson;.

b Teste exato de Fisher;.

c média; n - número total de pacientes inclusos; RENDA per capita = renda familiar/número de pessoas na casa em reais.

Os resultados das características de saúde, hábitos de vida, acesso ao serviço de saúde e motivos de abandono do tratamento de hanseníase coletados dos participantes são apresentados na Tabela 2. Verificou-se que, entre as pessoas com hanseníase que abandonaram o tratamento, 42,5% já haviam retomado a terapêutica, assim como 42,5% pretendiam retomar e 15% não pretendiam. Diante disso, foram realizados análise de associação entre as demais variáveis e o desfecho "continua sem retornar ao tratamento". A análise bivariada demonstrou como fator de risco para continuar sem tratamento ser sexo masculino, possuir dúvida sobre o tratamento e/ou doença, presença de diabetes, não ter melhora de sintomas após início de tratamento, e a condiação de ter outro médico descartando o diagnóstico de hanseníase/não acreditar ter a doença.

**Tabela 2:** Distribuiação de frequência das características de saúde, hábitos de vida, acesso ao serviço de saúde e motivos de abandono do tratamento de hanseníase e associação com a não adesão ao tratamento medicamentoso para hanseníase no período de 2017-2018, Palmas, Tocantins, Brasil (N=40).

Variáveis	n(%)	Continua sem retornar ao tratamento		Valor de *p*
		Sim (%) (n=23)	Não (%) (n=17)	
Tabagismo				
Sim	7(17,5)	3(42,8)	4(57,2)	1,000^b^
Não	33(82,5)	19(57,6)	14(42,4)	
Uso de álcool				
Sim	24(60)	13(54,2)	11(45,8)	0,601^a^
Não	16(40)	10(62,5)	6(37,5)	
Hipertensão arterial				
Sim	13(32,5)	3(23)	10(77)	0,103^b^
Não	27(67,5)	14(51,8)	13(48,2)	
Diabetes				
Sim	6(15)	6(100)	0(0)	**0,030** ^b^
Não	34(85)	17(50)	17(50)	
Obesidade				
Sim	2(5)	2(100)	0(0)	0,499^b^
Não	38(95)	21(55,3)	17(44,7)	
Dificuldade de acesso ao serviço de saúde?				
Sim	5(12,5)	2(40)	3(60)	0,634^b^
Não	35(87,5)	21(60)	14(40)	
Recebeu orientação sobre doença/tratamento?				
Sim	38(95)	21(55,3)	17(44,7)	0,499^b^
Não	2(5)	0(0)	2(100)	
Possui dúvida sobre doença/tratamento?				
Sim	17(47,5)	2(11,7)	15(88,3)	**0,001** ^b^
Não	23(57,5)	15(65,2)	8(34,8)	
Melhorou sintomas após início tratamento?				
Sim	24(60)	8(33,3)	16(66,7)	**0,000** ^b^
Não	16(40)	15(93,7)	1(6,3)	
Reação medicamentosa?				
Sim	27(67,5)	11(40,7)	16(59,3)	0,746^a^
Não	13(32,5)	6(46,2)	7(53,8)	
Recebeu visita de agente comunitário de saúde?				
Sim	38(95)	21(55,3)	17(44,7)	0,212^b^
Não	2(5)	2(100)	0(0)	
Relatou sobre a doença para outros?				
Sim	33(82,5)	20(60,6)	13(39,4)	0,432^b^
Não	7(17,5)	3(42,8)	4(57,2)	
Sentiu-se discriminado?				
Sim	21(52,5)	12(57,2)	9(42,8)	0,962^a^
Não	19 (47,5)	11(57,9)	8(42,1)	
Por que desistiu do tratamento?				
Outro médico descartou diagnóstico/não acredita ter a doença	20(50)	14(73,7)	5(26,3)	**0,049** ^a^
Reação a medicação/uso de álcool/outros motivos	20(50)	9(42,8)	12(57,2)	

a Teste qui-quadrado de Pearson;.

b Teste exato de Fisher; n - número total de pacientes inclusos.

O modelo de análise multivariada demonstrou que as variáveis "não ter melhora dos sinais e sintomas" e "ter o diagnóstico de hanseníase descartado por outro médico" e/ou "não acreditar na doença" foram fatores significantes para continuar sem tratamento de hanseníase (Tabela 3).

**Tabela 3:** Modelo de regressão de Poisson robusta para estimar as razões de prevalência dos fatores associados ao continuar sem o tratamento de hanseníase no período de 2017-2018, Palmas, Tocantins, Brasil (N=40).

Variáveis	Modelo inicial	Valor de *p*	Modelo final	Valor de *p*
	RP (IC95%)		RP (IC95%)	
Sexo				
Masculino	0,94(0,53-1,67)	0,856	-	-
Feminino	1		-	-
Ocupação				
Autônomo/administrativo	1		-	-
Aposentado/desempregado	1,70(1,05-2,75)	0,028	-	-
Diabetes				
Não	1		-	-
Sim	1,13(0,74-1,73)	0,551	-	-
Dúvida sobre a doença/tratamento				
Não	1		1	
Sim	1,84(1,10-3,08)	0,020	1,93(1,10-3,39)	0,02
Melhora dos sintomas				
Sim	1		1	
Não	1,92(1,14-3,23)	0,013	2,25(1,26-4,04)	0,00
Motivo de desistência do tratamento				
Outro médico descartou diagnóstico/não acredita ter a doença	1,44(0,88-2,36)	0,141	-	-
Reação a medicação/uso de álcool/outros motivos	1		-	-
Constante	0,11(0,03-0,38)	0,000	0,26(0,14-0,47)	0,000
Pseudo R^2^	13,6%		11,3%	
Critério de Informação de Akaike	75,733		69,374	
Critério de Informação Bayesiano	87,555		74,441	

RP - razão de prevalência; IC95% - intervalo de confiança de 95%.

A Tabela 4 demonstra que 37,5% dos participantes relatam necessidade de melhora do serviço, sendo o atendimento/recepação o setor mais mencionado (25%). Entre os 23 participantes que continuam sem retornaram ao tratamento, apenas seis pessoas relataram pretensão de retomar, sendo o principal impedimento citado para esse retorno o relato de se considerar curado e não ter mais a doença (42,5%).

**Tabela 4:** Características quanto ao serviço de saúde e não adesão dos pacientes identificados pelo Sistema de Informação de Agravos de Notificação como casos de abandono de tratamento de hanseníase no período de 2017-2018, Palmas, Tocantins, Brasil (N=40).

Características	n(40)	%
Identifica a necessidade de melhora do serviço?		
Sim	15	37,5
Não	25	62,5
O que precisa melhorar no serviço de saúde?		
Atendimento/recepação	10	25
Mais profissionais médicos	5	12,5
Não se aplica	25	62,5
Pretende retomar o tratamento?		
Sim	17	42,5
Não	6	15
Já retornou	17	42,5
Qual é seu impedimento para retomar o tratamento?		
Relata não ter doença ou estar curado	17	42,5
Outros (uso de álcool, reação medicamento, dificuldade de acesso)	6	15
Não se aplica	17	42,5

n - número total de pacientes inclusos; % - porcentagem.

## DISCUSSÃO

A hanseníase apresenta indicadores de incidência elevados na região Norte do país, especificamente no estado do Tocantins, sendo considerada uma região hiperendêmica com transmissão ativa e contínua^(15,16)^. Regiões brasileiras com as maiores cargas de hanseníase (regiões Norte, Nordeste e Centro-Oeste do Brasil) têm maiores chances de abandono do tratamento em relação à s regiões como Sul e Sudeste, com menor carga^(7)^.

Nesse contexto, o abandono do tratamento na hanseníase representa um dos desafios mais relevantes ao controle de doenças infecciosas crônicas que requerem tratamento de longo prazo^(17)^. Estudo utilizando modelagem matemática demonstrou que a não adesão à PQT e o abandono podem ter um impacto negativo na erradicação da hanseníase, levando a um aumento na prevalência da doença e mortes relacionadas em todo o mundo^(18)^.

Nosso estudo demonstra taxas de abandono de 8,3% e 3,12% nos anos de 2017 e 2018 respectivamente, sendo superiores ao encontrados em outros estudos^(7,10,17)^. Os resultados desta pesquisa indicam que a falta de melhora dos sintomas e o descarte do diagnóstico por outro médico, assim como a não crença na presença da doença, são barreiras à adesão ao tratamento da hanseníase. Além disso, a análise bivariada revelou que o sexo masculino, a presença de diabetes e dúvidas sobre o tratamento estão associados ao não retorno à PQT.

Destaca-se a escassez de estudos publicados que investigam os fatores associados ao abandono do tratamento da hanseníase^(7,8,10,17,19)^, sendo que a maioria não explora características como motivo de abandono, acesso e qualidade do serviço de saúde, orientações sobre a doença, dúvidas e discriminação, como feito neste estudo. Ressalta-se o desafio de execuação de pesquisas como esta, diante das dificuldades de busca e encontro dos casos de abandono, como mencionado em outros estudos^(8)^.

A melhora dos sintomas da hanseníase já é conhecida como condiação que favorece ao abandono do tratamento da hanseníase^(7,8)^. Entretanto, apesar de 60% dos participantes referirem melhora dos sintomas após uso da PQT, nossos resultados demonstram que a não melhora dos sintomas é que estaria relacionada ao não retorno ao tratamento. Esse resultado sugere a necessidade de estudos mais robustos nesse campo que abordem fatores qualitativos relacionados ao abandono do tratamento pelo paciente de hanseníase, como a aceitação da doença, e aspectos de domínios psíquico, social e cultural.

Em 73,7% dos casos de abandono que permaneceram sem tratamento, indica-se como motivo de desistência o descarte do diagnóstico por outro médico (geralmente da rede particular) ou a falta de crença na presença da doença. A ausência de estudos que demonstrem tal resultado demanda reflexão, bem como a realização de pesquisas futuras para investigar essa questão mais profundamente.

Pesquisas qualitativas sobre o abandono do tratamento da hanseníase destacam a fragilidade na confiabilidade por parte do paciente em relação ao diagnóstico médico e à falta de aceitação do uso da PQT como ferramenta para obtenação da cura da doença^(20,21)^. Sabe-se que a hanseníase é uma patologia que carrega muito estigma. Dessa maneira, muitos pacientes, por apresentarem sentimentos negativos em relação à doença, acabam não aceitando um diagnóstico inicial, optando por buscar opinião de outro profissional, mas este, por sua vez, pode apresentar certo despreparo e descartar o diagnóstico^(22)^. Pessoas com suspeita de hanseníase acabam por percorrem uma longa jornada em busca de uma resposta sobre seu estado de saúde, e, muitas vezes, recebem um diagnóstico equivocado que pode gerar graves consequências futuras^(23)^.

Mesmo conhecendo a patologia, alguns acometidos podem rejeitar a ideia de serem portadores da doença e relatarem não sentir nenhum tipo de sintoma^(21)^. Acredita-se que a intensidade do sentimento de não aceitação por parte dos pacientes possa dificultar ainda mais o processo de adesão ao tratamento, principalmente em pacientes que não apresentam sintomas característicos da hanseníase, aumentando o descontentamento em relação ao diagnóstico devido a conceitos preconceituosos internalizados. Todos os problemas psíquicos ou sociais causados pela doença são inerentes ao preconceito, discriminação e estigma existentes no indivíduo e na sociedade^(24)^.

Fica evidente que a manifestação da negação da enfermidade é condiação presente em grande parte dos pacientes, por ainda ser uma doença estigmatizada. A negação é caracterizada por um mecanismo de defesa no qual a realidade é negada como forma de evitar sofrimento, seja ele físico ou psíquico. Nesse aspecto, é de extrema importância para a equipe de profissionais considerar essas manifestações desde o diagnóstico e realizar ao acompanhamento do paciente, pois a adesão ao tratamento, mudança no estilo de vida e comportamento partem do princípio que o paciente aceita sua condiação e se torna agente corresponsável pela recuperação de sua saúde^(20-22)^.

Houve predominância do sexo masculino em 61,25% dos casos de abandono nos anos de 2017 e 2018, porém, após a busca ativa para coleta de pesquisa, a amostra foi constituída por maioria do sexo feminino (57,5%), demonstrando a dificuldade em alcançar e identificar indivíduos do sexo masculino em situação de abandono do tratamento da hanseníase. Estudos apontam a maior resistência dos homens a procurar os serviços de saúde^(8,10)^, acarretando menos orientação sobre a doença e interferindo na adesão e seguimento do tratamento^(25)^.

No que se refere aos aspectos de cor/etnia, sabe que a hanseníase pode atingir qualquer indivíduo. Entretanto, os indicadores de saúde no Brasil, com base nessa variável, têm apontado que negros e pardos apresentam números maiores quando comparados à população branca^(7)^. Tal fato pode ser resultado de desigualdades sociais ainda existentes no país, necessitando de estudos mais afins para entender a respeito da distribuiação neste grupo e fatores associados^(7,26)^. Destaca-se que o estado do Tocantins possui maior proporação de negros e pardos em relação à s outras categorias^(11)^.

Os resultados demonstram 60% dos participantes tendo até ensino fundamental, sendo 10% desses analfabetos, evidenciando baixo grau de escolaridade entre os casos de abandono, assim como demonstrado em outras pesquisas^(7,10)^. Esses achados podem configurar limitações no processo de compreensão da patologia e seu tratamento por parte dos pacientes, desencadeando maior resistência na adesão do tratamento, seguimento adequado e episódios de abandono da PQT.

O uso de álcool foi evidenciado por 60% dos participantes deste estudo, dos quais 15% relataram consumir bebidas alcoólicas diariamente, caracterizando dependência. Além de ser um problema mundial que influencia comportamentos e prejudica a saúde dos afetados, o consumo de álcool é um fator que interfere negativamente no processo de aceitação da doença e no uso da medicação. Ele também pode diminuir o efeito curativo dos medicamentos e ser um fator preditivo para a recidiva da doença^(10,27)^.

A correlação entre álcool e abandono do tratamento já é reconhecida^(7,28)^, pois o álcool pode levar ao esquecimento na tomada das medicações diárias ou ser contraindicado durante tratamentos médicos. Consequentemente, algumas pessoas podem optar por continuar consumindo álcool, mesmo em detrimento à terapêutica, seja devido a um nível social e educacional desfavorável (que dificulta o reconhecimento da importância do tratamento) ou à dependência química^(7)^. Nesse contexto, observa-se a necessidade de uma abordagem multiprofissional e intersetorial para o manejo e acompanhamento desses pacientes, especialmente o apoio da rede de atenação especializada para pacientes em uso abusivo de álcool e drogas.

A apresentação de comorbidades em pessoas com hanseníase pode potencializar o não seguimento correto do tratamento medicamentoso, pois o uso concomitante de várias medicações diárias pode deixar o paciente confuso quanto ao uso e ocasionar mal-estar devido a interações medicamentosas, tornando-o mais vulnerável ao abandono^(10,29)^. Neste estudo, 100% das pessoas com diabetes e hanseníase persistiram sem retornar ao tratamento de hanseníase.

Entende-se que, no momento do diagnóstico de qualquer doença, é necessário que a equipe de profissionais ofereça apoio ao paciente e realize as orientações adequadas quanto ao processo da doença e seu tratamento, de forma que ele não tenha dúvidas, e, assim, possa ser protagonista no reestabelecimento de sua saúde. Sabendo da relação proporcional entre informação e abandono do tratamento^(7)^, neste estudo, 95% relataram receber orientações sobre a hanseníase e seu tratamento no momento do diagnóstico, porém 42,5% referiram ainda ter dúvidas sobre a doença, assim como seu tratamento. Essa variável foi associada, significantemente, ao não retorno ao tratamento de hanseníase na análise bivariada. Assim, alguns questionamentos podem ser levantados quanto aos processos de educação em saúde realizados pelas equipes de saúde do município, principalmente relacionados à qualificação dos membros da equipe de saúde, ao conhecimento prévio dos pacientes sobre a doença, à inclusão da família no tratamento, entre outros.

Essa fragilidade pode estar associada ao desconhecimento de muitos profissionais sobre a doença, o tratamento e seus efeitos adversos, limitando, assim, o processo de educação em saúde ou ainda pode estar relacionado a uma orientação realizada em linguagem inapropriada, não considerando o nível de compreensão do paciente^(23)^. Diante disso, destaca-se a importância de ações de educação permanente para melhoria da qualidade assistencial no enfretamento clínico da hanseníase. Também se faz necessário o estabelecimento de um relacionamento terapêutico entre profissional e paciente, trabalhando aspectos de empatia e holisticidade nos momentos das consultas, principalmente na ocasião do diagnóstico, considerando os conhecimentos prévios, escolaridade, crenças, medos e inseguranças, auxiliando para um melhor entendimento e adesão ao tratamento^(30)^.

Nesta pesquisa, 52,5% referiram discriminação em algum momento pós-diagnóstico. A hanseníase é uma doença carregada de distorções, estigmas e preconceitos que intervêm diretamente na adesão ao tratamento, causando situações conflituosas no seio familiar e social, além de promover sofrimento psíquico ao paciente, interferindo nas atividades diárias, trabalho e na sua qualidade de vida. Em condições mais graves, leva à perda da autoestima e depressão, em que muitas vezes os serviços primários de saúde não conseguem oferecer um cuidado satisfatório, levando essas pessoas ao isolamento. Dessa forma, percebe-se a necessidade de aprimorar habilidades e percepções por parte dos profissionais quanto ao manejo integral e de qualidade da hanseníase^(31)^.

É importante destacar as fragilidades nos processos de trabalho das equipes de saúde na assistência à s pessoas com hanseníase, como a "perda" dos casos na rede de saúde, o que resulta na descontinuidade do cuidado. A realização deste estudo demonstra que, mesmo com a busca ativa, não foi possível identificar a localização de 53% dos casos de abandono de hanseníase. Esse cenário evidencia a importância da atenação primária como ordenadora do cuidado na rede, acompanhando a trajetória terapêutica dessas pessoas dentro da rede e atuando na busca ativa dos casos de abandono.

Da mesma forma, os casos de abandono no tratamento da hanseníase apontam para a importância do acompanhamento por visitas domiciliares do agente comunitário de saúde, trabalho em equipe, orientações sobre as sequelas permanentes e treinamento dos profissionais orientado pela prática, entre outros^(32)^.

Os efeitos adversos ocasionados pela PQT podem ocasionar desconforto físico diário aos usuários, frequentemente associados à resistência na adesão ao tratamento e, em muitos casos, ao abandono. Este estudo demonstra que 25% dos pacientes participantes do estudo abandonaram o tratamento da hanseníase devido à reação aos medicamentos. O Ministério da Saúde, objetivando combater as reações adversas e intolerância medicamentosa desenvolvidas durante o tratamento, estabeleceu protocolos de substituiação de esquemas, objetivando dirimir os efeitos indesejáveis da PQT^(26)^. Evidencia-se também a resistência antimicrobiana, frequentemente relatada como consequência dos episódios de abandono terapêutico^(25)^. Dessa forma, destaca-se o papel dos profissionais de saúde para realizar o acompanhamento dos pacientes em tratamento diante dos efeitos colaterais, procurando oferecer apoio e avaliando a necessidade de estabelecer outro esquema alternativo preconizado, no intuito de conduzir um tratamento com menos efeitos indesejáveis, promovendo uma melhor qualidade de vida no seguimento do regime terapêutico.

### Limitações do estudo

É importante reconhecer as limitações inerentes a esta pesquisa, tais como o número de casos de abandono de tratamento devido a dificuldades operacionais, como a localização dos usuários (seja por mudança de endereço ou endereço incompleto no SINAN e no prontuário). Além disso, devido à natureza transversal do estudo, não foi possível estabelecer fatores causais, assim como a singularidade do local pesquisado, que pode não refletir a realidade de outros locais do país. Nesse sentido, recomendamos a realização de outras pesquisas utilizando metodologias que permitam identificar outros possíveis fatores relacionados à não adesão ao tratamento da hanseníase.

### Contribuições para as áreas da enfermagem, saúde ou políticas públicas

O estudo contribui para identificação de fatores envolvidos na situação de abandono do tratamento PQT da hanseníase em uma capital hiper endêmica do norte do país, destacando variáveis não descritas anteriormente, como a não melhora dos sintomas, ter o diagnóstico descartado por outro médico e não acreditar ter a doença, como os mais relevantes.

## CONCLUSÕES

Os fatores associados à não adesão ao tratamento PQT para hanseníase, identificados pela análise bivariada neste estudo, foram sexo masculino, dúvidas sobre o tratamento e/ou a doença, presença de diabetes, ausência de melhora nos sintomas após o início do tratamento, bem como o fato de outro médico ter descartado o diagnóstico de hanseníase ou a pessoa não acreditar ter a doença. Após a análise multivariada, observou-se que a ausência de melhora nos sinais e sintomas e o diagnóstico de hanseníase descartado por outro médico ou a falta de crença na doença se mostraram significativos para o modelo. O estudo revela achados importantes relacionados à não adesão ao tratamento de hanseníase, fornecendo subsídios para a implementação de ações e estratégias que melhorem a qualidade do cuidado, além de orientar pesquisas futuras que visem compreender de forma mais aprofundada os fatores intrínsecos, bem como aqueles relacionados ao serviço e à assistência prestada à s pessoas com hanseníase.

## Data Availability

Os dados da pesquisa estão disponíveis no corpo do artigo.
